# Body, territory and memory: midwifery in Afro-descendant communities. Colombia

**DOI:** 10.15649/cuidarte.5275

**Published:** 2026-09-15

**Authors:** Yasnury Vanegas-Moreno, Claudia Alvarez-Franco

**Affiliations:** 1 Master’s degree in Collective Health. Universidad de Antioquia, Medellín, Colombia. E-mail: yasnury.vanegas@udea.edu.co Universidad de Antioquia Medellín Colombia yasnury.vanegas@udea.edu.co; 2 PhD in Nursing. Full Professor, Faculty of Nursing. Universidad de Antioquia; Medellín, Colombia. E-mail: claudia.alvarez@udea.edu.co Universidad de Antioquia Medellín Colombia claudia.alvarez@udea.edu.co

**Keywords:** Midwifery, African-American Traditional Medicine, Childbirth, Parteras Tradicionales, Medicina Tradicional Afrodescendiente, Nacimientos en Casa, Tocologia, Medicina Tradicional Afro-Americana, Partos Domiciliares

## Abstract

**Introduction:**

This article analyzes the meanings and practices of traditional Afro-descendant midwives in Nuquí, Chocó, from a critical social and decolonial feminist perspective.

**Objective:**

To recognize their practices, knowledge, and challenges they face in relation to their territory, bodies, and community.

**Materials and Methods:**

A qualitative study was conducted using a critical ethnographic approach, participant observation, semi-structured interviews, and “circles of the word” (talking circles), prioritizing horizontal dialogue and epistemological reciprocity.

**Results:**

The findings were organized into three analytical categories. Practice and situated knowledge showed that midwives develop their knowledge through experience, ancestral heritage, and everyday observation, integrating care with community ties and intergenerational knowledge transmission. The legitimization of knowledge and grassroots power revealed tensions with the biomedical system, characterized by its institutional failure to recognize these practices. Finally, free bodies and knowledges of the land highlighted a profound connection among body, territory, and spirituality, in which the body is understood as a sacred territory, and health is linked to the use of medicinal plants, harmony with nature, and collective rituals.

**Discussion:**

Midwives’ knowledge represents forms of care rooted in non-hegemonic worldviews, whose marginalization reflects epistemic injustice sustained by colonial and patriarchal logics.

**Conclusion:**

It is urgent to recognize and support midwives and ensure their role receives dignified recognition through intercultural public policies that guarantee training, support, and fair compensation, as a pathway toward epistemic and cultural justice.

## Resumen

**Introducción:**

Este artículo analiza los significados y prácticas de las parteras tradicionales afrodescendientes de Nuquí, Chocó, desde una perspectiva crítica social y feminista decolonial.

**Objetivo:**

Reconocer las prácticas, saberes y desafíos que enfrentan en relación con su territorio, cuerpo y comunidad.

**Materiales y métodos:**

Se desarrolló una investigación cualitativa con enfoque de etnografía crítica, mediante observación participante, entrevistas semiestructuradas y círculos de palabra, priorizando el diálogo horizontal y la reciprocidad epistemológica.

**Resultados:**

Los hallazgos se organizaron en tres categorías analíticas: en Práctica y conocimiento situado, se evidenció que las parteras construyen su saber desde la experiencia, la herencia ancestral y la observación cotidiana, articulando el cuidado con vínculos comunitarios y la transmisión intergeneracional del conocimiento; en Legitimación de saberes y poder popular, se identificaron tensiones con el sistema biomédico, marcado por la invisibilización institucional de sus prácticas; por último, en Cuerpos libres y saberes de la tierra, emergió una profunda conexión entre cuerpo, territorio y espiritualidad, donde el cuerpo es concebido como territorio sagrado y la salud se vincula al uso de plantas medicinales, la armonía con la naturaleza y los rituales colectivos.

**Discusión:**

El saber de las parteras representa formas de cuidado arraigadas en cosmovisiones no hegemónicas, cuya marginalización refleja una injusticia epistémica sostenida por lógicas coloniales y patriarcales.

**Conclusiones:**

Es urgente reconocer, apoyar y dignificar el reconocimiento de las parteras, mediante políticas públicas interculturales que garanticen formación, apoyo y remuneración digna, como camino hacia la justicia epistémica y cultural.

## Resumo

**Introdução:**

Este artigo analisa os significados e práticas de parteiras afrodescendentes tradicionais em Nuquí, Chocó, a partir de uma perspectiva feminista crítica social e decolonial.

**Objetivo:**

Reconhecer as práticas, o saber e os desafios que elas enfrentam em relação ao seu território, corpo e comunidade.

**Materiais e métodos:**

Foi realizado um estudo qualitativo utilizando uma abordagem etnográfica crítica, empregando observação participante, entrevistas semiestruturadas e círculos de discussão, priorizando o diálogo horizontal e a reciprocidade epistemológica.

**Resultados:**

Os achados foram organizados em três categorias analíticas: em Prática Situada e Saber, evidenciou-se que as parteiras constroem seu saber a partir da experiência, da herança ancestral e da observação diária, articulando o cuidado com os laços comunitários e a transmissão intergeracional do saber; em Legitimidade do Saber e Poder Popular, foram identificadas tensões com o sistema biomédico, marcadas pela invisibilidade institucional de suas práticas; Por fim, em Corpos Livres e o Conhecimento da Terra, emergiu uma profunda conexão entre corpo, território e espiritualidade, onde o corpo é concebido como território sagrado e a saúde é associada ao uso de plantas medicinais, à harmonia com a natureza e a rituais coletivos.

**Discussão:**

O saber das parteiras representa formas de cuidado enraizadas em cosmovisões não hegemônicas, cuja marginalização reflete uma injustiça epistêmica sustentada por lógicas coloniais e patriarcais.

**Conclusões:**

É urgente reconhecer, apoiar e dignificar o trabalho das parteiras por meio de políticas públicas interculturais que garantam formação, apoio e remuneração justa, como um caminho para a justiça epistêmica e cultural.

## Introduction

Traditional midwifery has historically been a cornerstone of maternal and child health care. In the Afro-descendant communities of Nuquí, Chocó, it was recognized by the Colombian government through Resolution 1077 of 2016^1^. Afro-Pacific midwifery is far more than a medical practice; it embodies a comprehensive system of care grounded in ancestral knowledge, spirituality, and a profound relationship with nature. However, despite this regulatory recognition, it continues to face stigmatization and exclusion in healthcare and legal settings. This practice is deeply rooted in a worldview that integrates spirituality, ancestral knowledge, and respect for nature within a territory located in the western part of the department of Chocó, on the western slope of the Baudó mountain range and along the Pacific Ocean^2^. Afro-descendant midwives not only provide support throughout childbirth but also safeguard a cultural heritage expressed through rituals, practices, and knowledge transmitted from generation to generation. This heritage is closely connected to an environment characterized by high biodiversity, abundant water resources, and territorial dynamics that shape community ways of life, production, and care^3^.

According to information provided by the municipality, Nuquí is located in the western part of the department of Chocó, on the western slope of the Baudó mountain range, along the Pacific Ocean. The municipal seat is situated at sea level and borders other localities in Chocó and the Pacific Ocean. The municipality covers approximately 956 km², most of which is rural and characterized by high rainfall, a dense network of waterways, and biodiversity that directly shapes residents’ ways of life, production, and care^4^,^5^. From a critical and decolonial perspective, this study seeks to bring visibility to and affirm the role of midwives as central figures in their communities, while also contributing to the safeguarding of their practices, promoting intercultural dialogue, and advocating for public policies that recognize midwifery as a legitimate, vital, and profoundly human body of knowledge. Its objective was to identify the practices, knowledge, and challenges midwives face in relation to their territory, bodies, and community.

## Materials and Methods

Research approaches make it possible to understand a phenomenon from ontological and epistemological perspectives and provide the basis for methodological decisions and validity criteria^6^. In this study, qualitative research enabled the exploration of subjective dimensions (memory, emotions, and culture)^7^,^8^. Denzin and Lincoln caution that the use of decontextualized techniques may reinforce structures of control^9^. Fricker introduced the concept of epistemic injustice to describe the exclusion of subaltern forms of knowledge from knowledge production^10^. In this context, Black feminism and intersectionality advance a situated epistemology that challenges universalizing narratives and brings visibility to the intersecting forms of oppression experienced by racialized women^11^,^12^. These theoretical and political perspectives constitute ethical commitments to resistance and epistemic justice.

From a methodological standpoint, a critical ethnographic approach was adopted. Unlike traditional ethnography, critical ethnography acknowledges the researcher’s influence on the construction of knowledge and promotes reflexive practice that questions the researcher’s own assumptions^13^,^14^. In contexts such as that of the midwives of Nuquí (Chocó), this methodology fosters ethical dialogue with local knowledge systems and recognizes their voices and practices as legitimate forms of knowledge^15^.

The research was conducted with a group of eight midwives from the municipality of Nuquí, Chocó. Purposive sampling was used, prioritizing women recognized for their community leadership and extensive experience in traditional midwifery. Participants were contacted through local women leaders and community networks. A total of 16 interviews were conducted from late 2023 through 2024, each lasting between 60 and 120 minutes. These comprised 10 semistructured interviews and six in-depth interviews, in addition to two“circles of word”(talking circles) and participant observation. The interviews took place in the midwives’ everyday settings, including their homes and community spaces, without third parties present, and were audio-recorded after obtaining prior informed consent.

A flexible topic guide was used and refined following an initial pilot phase conducted with women from Chocó living in Medellín who were knowledgeable about traditional midwifery. The prepared questions were administered to these women and subsequently revised. The analysis incorporated the participants’ voices, with the categories validated and expanded through reflective dialogue; the co-construction of knowledge was a central component of the study.

The analysis was conducted manually and collaboratively by the two researchers, following the principles of qualitative critical ethnographic methodology^16^. A data systematization matrix was developed to organize interview excerpts, from which subcategories were derived and subsequently grouped into categories, taking into account the theoretical perspectives underpinning the study. The researchers’ prior experience in qualitative research, gender, interculturality, and community-based work also contributed to a sensitive, reflexive, and respectful approach to the narratives and experiences shared by the participants. A triangulation process integrating interviews, observations, field notes, and images was also conducted, thereby strengthening the credibility of the interpretations until saturation was reached. Verbatim quotations were included in the findings to ensure transparency and fidelity to the participants’ discourse. In addition, selected interpretations and emerging categories were discussed with the participants to expand, compare, and collectively validate the findings. This article follows the reporting criteria for qualitative research described in the Consolidated Criteria for Reporting Qualitative Research (COREQ) checklist.

This study adhered to ethical principles grounded in the Ubuntu philosophy, which promotes harmony among people, life, and nature^17^, as well as to international standards, including the Declaration of Helsinki^18^ and the Universal Declaration on Bioethics and Human Rights, and national regulations, including Statutory Law 1581 of 2012^19^. Resolution 8430 of 1993^20^, and Resolution 40455 of 2015, as amended by Resolution 47765 of 2021^21^. These principles and regulations informed the informed consent process for audio recording and image capture. The study was approved by the ethics committee, as documented in Minutes No. 81CEI-FE_2023. The researchers were trained women, which contributed to a sensitive and respectful approach to the participants’ accounts.

The complete dataset is openly available for access and consultation through Mendeley Data^22^.

## Results

The findings, organized into three categories, emerged from research on Afro-descendant midwifery in Colombia’s Pacific region conducted as part of the researcher’s master’s studies. These categories describe how this body of knowledge is constructed, legitimized, and sustained in contexts of cultural and territorial diversity. The first category, “practice and situated knowledge,” explores how midwives acquire and apply their knowledge in specific settings.

The second category, “legitimation of knowledge and grassroots power,” addresses tensions between institutional health systems and traditional knowledge. The third, “free bodies and knowledge of the land,” examines the relationship among bodily care, nature, and autonomy. Each category includes subthemes that provide a detailed understanding of the dynamics shaping midwifery practice from ancestral and territorial perspectives.

**Practice and situated knowledge**

In Nuquí, midwifery is an ancestral practice transmitted orally across generations, as shown in Figure 1. More than a technique, it is a living art connected to territory, memory, and family ties. Many midwives reported learning from their mothers or grandmothers: “I have 36 years of experience as a midwife, and I learned from my mother” (OG1). This account reflects a form of learning grounded in observation and support. Elder midwives are central figures who transmit knowledge and sustain community networks (OG2).

**Figure 1:**
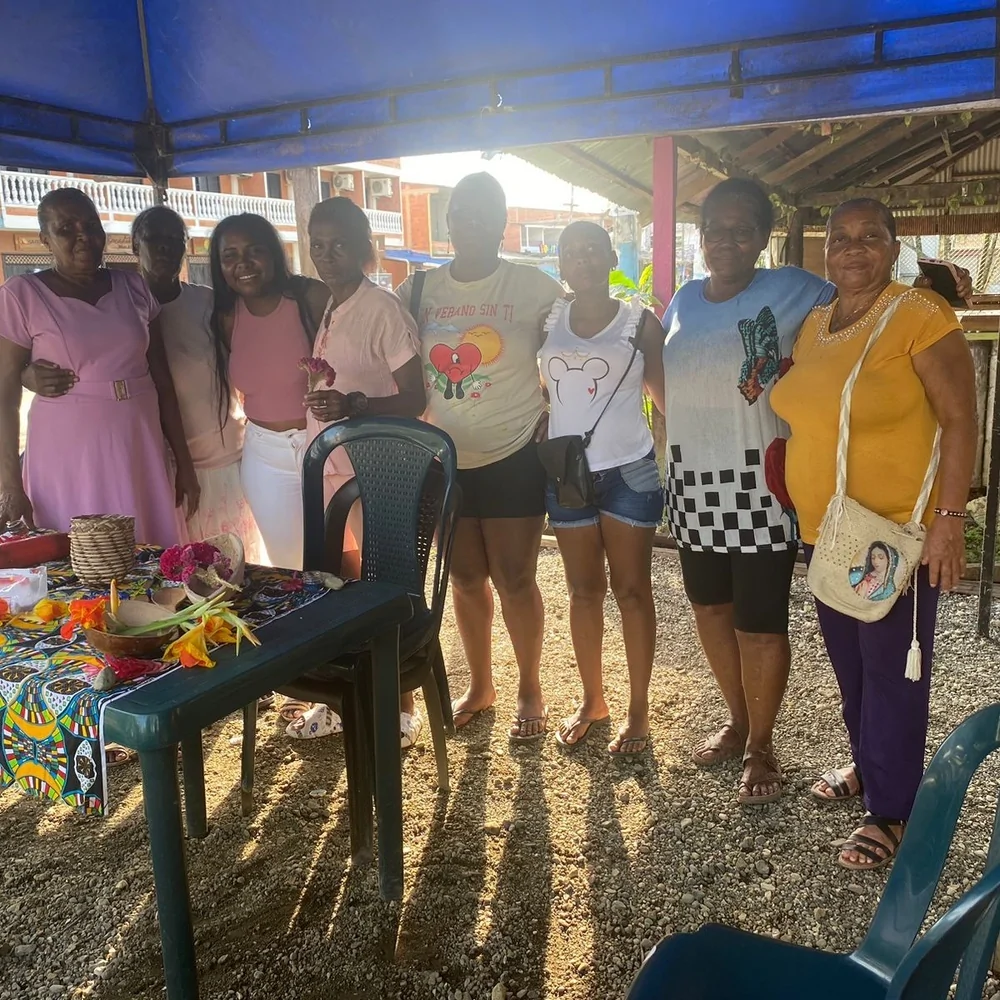
Group of traditional midwives from Nuquí

*Note: Photograph taken by the author in Nuquí (2024)*

However, learning is not limited to the maternal lineage. Some women entered midwifery out of necessity, such as one midwife who attended the birth of twins at age 19 in Juradó (PM1). These experiences shape a collective form of care and resistance.

Being a midwife is a source of pride, identity, and commitment to life. “I am saving another life… midwives are essential to the municipality” (IV11). However, this work is carried out under precarious conditions, including barriers to access, limited resources, and inadequate infrastructure. “They came to get me by river… the woman was giving birth” (AM2), one midwife recalled, illustrating the geographic and logistical challenges they face. Other midwives expressed a desire for appropriate facilities: “I would like to have a place with a proper bed” (DM1).

In response to these limitations, midwives combine ancestral knowledge with intuition and creativity. They use medicinal plants in situations in which the biomedical system is unable to respond: “When the doctors cannot stop the bleeding, I use chicory, and it works” (DM4). Although they recognize the limits of their knowledge, they also understand when referral to a health care facility is necessary: “When childbirth becomes risky… the woman has to be taken to the health center” (OG12).

Thus, midwives sustain their practice through wisdom, resilience, and love of life. Their work, which is essential in contexts of exclusion, represents a form of resistance to institutional neglect and an affirmation of the sovereignty of ancestral knowledge in community care

**Free bodies and knowledge of the land**

Traditional midwifery is sustained by a profound relationship among women, the body, and the territory. Within this interconnected framework, nature is not an external resource but a vital ally and a tapestry of wisdom that accompanies midwives in their everyday practice. The alliance between midwives and nature is a central pillar of ancestral knowledge that has endured across generations. This connection with the land and its elements is expressed particularly through the use of medicinal plants. Over time, midwives have learned to interpret signs in nature and to use the resources it provides to support women through the process of giving birth, as one participant explained: “Well, yes, in the past, women had their little secrets, but now many ideas have been taken from us. We even share knowledge with Indigenous women, and they share with us” (OG16). This account highlights the collective and dynamic nature of this knowledge, which is enriched through dialogue among communities, such as that occurring between Afro-descendant and Indigenous women.

This knowledge is not activated only during critical moments in childbirth; rather, it is part of the midwives’ everyday lives. “And it is different: if I tell her, ‘This herb can be crushed to induce vomiting,’ she uses it for something else, and another woman uses it for yet another purpose” (IV2). This statement illustrates how botanical knowledge is exchanged and enriched among women, weaving a network of knowledge that strengthens both bodily care and the relationship with the natural environment.

Maintaining this connection with the land is also an act of resistance to medicalization and the imposition of hegemonic health models. When midwives draw on natural remedies and traditional techniques, they not only provide comprehensive care but also defend a way of life grounded in balance between the body and nature. Through these practices, they protect women’s health and safeguard a heritage that has demonstrated its effectiveness and wisdom over time.

This vital alliance finds its fullest expression in the use of medicinal plants, which many midwives regard as the very heart of midwifery. The cultivation, harvesting, and use of these plants constitute an essential component of midwifery knowledge, as shown in Figure 2.

For many midwives, midwifery is not possible without plants, as one participant stated: “A midwife who does not even have nacedera growing in her yard is not a midwife, because how is she going to prepare a little herbal drink for a woman? How is she going to help her?” (OG17). This account illustrates that knowledge of medicinal plants is not merely complementary but a fundamental basis of midwifery practice. With the aid of infusions, baths, poultices, and herbal smoke treatments, midwives relieve pain, prepare the body for childbirth, and care for women during the postpartum period.

However, material conditions are not always favorable, and many midwives lose their medicinal gardens, as one participant recounted: “I don’t have one right now because a chicken came and destroyed everything, but you always keep a little plant somewhere and grow it in case it can help with something” (AM4). This account illustrates the resilience and creativity with which midwives keep their knowledge alive despite everyday challenges.

**Figure 2:**
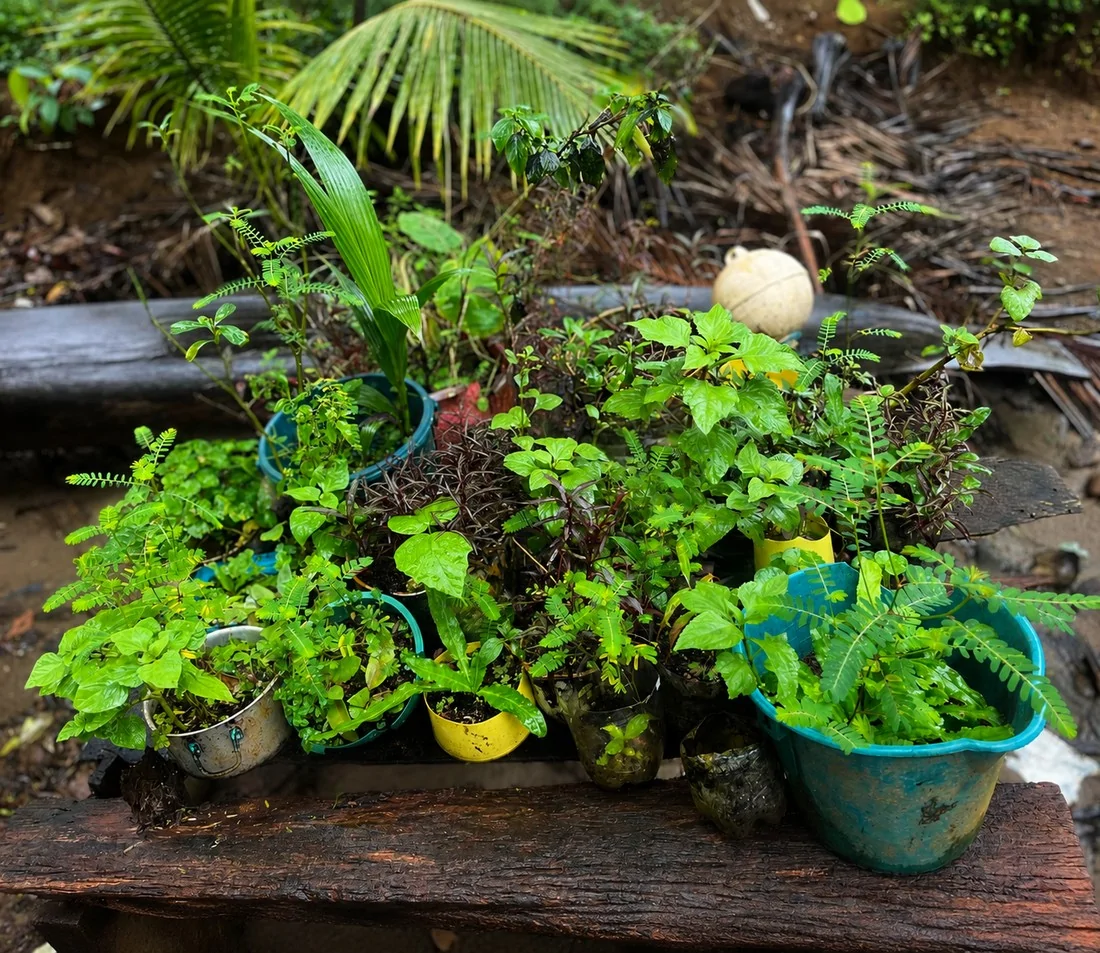
A midwife’s medicinal plant garden

*Note: Photograph taken by the author in Nuquí in 2024. Plants used during and after childbirth include basil (Ocimum basilicum L.), carpintero (pectoralis), descanse (Alternanthera bettzickiana), mugwort (Artemisia vulgaris), and nacedera (Trichanthera gigantea)*

Moreover, increasing medicalization has reduced the demand for these traditional practices. As another midwife lamented, “What has changed is that now people no longer come looking for us to attend births. You may have your herbs and know how to use them, but if they do not seek you out, how are you supposed to gather the plants and give them to the women?” (NM13). This shift threatens to silence knowledge that has long been a cornerstone of community health, displacing forms of care deeply connected to the land and spirituality.

Beyond their practical effectiveness, medicinal plants also hold symbolic and spiritual significance. They embody the connection between the human body and the territory, and between the visible and invisible dimensions of the birthing process. One midwife explained: “Every woman continues to bleed after giving birth, and the chicory stopped it all—and put a sack of salt on her” (DM2). Here, the plant not only acts on the physical body but also forms part of a ritual intended to provide protection and restore vital balance.

Ultimately, traditional midwifery, as practiced and experienced by these women, cannot exist without medicinal plants. Midwives are the guardians of a body of knowledge that not only safeguards health but also embodies a way of understanding and inhabiting the world. In the face of the pressures of modernity and institutional devaluation, they steadfastly maintain that without plants, there can be no midwifery. In doing so, they defend not only a practice but an entire worldview in which the body, the land, and care are woven into a single fabric of life.

**Legitimation of knowledge and grassroots power**

Integrating midwives’ knowledge into health systems not only enriches health care but also promotes intercultural collaboration in which scientific and community-based knowledge mutually reinforce one another. Far from posing a threat to formal medicine, traditional midwifery offers an opportunity to broaden approaches to care from a perspective rooted in culture, territory, and women’s dignity.

Training midwives in partnership with medical institutions, as has occurred in other countries, demonstrates that this knowledge can be legitimized without stripping it of its essence. This process is grounded in building relationships of respect and reciprocity in which midwives are recognized not only as health care providers but also as bearers of a profound cultural legacy. As one midwife explained:

“I dream of us being recognized, and of midwives in every community having a little place of our own where we could attend births—just a small room attached to the house where we could receive the woman, care for her, and then take her back home. I would really like that. It would be beautiful to see” (OG21).

This dream of having a private, dignified space of their own in which to care for women in their community encapsulates the desire to bring together cultural identity, autonomy, and respect for life. Beyond a physical setting, midwives are calling for an infrastructure that enables them to practice their knowledge with the necessary resources, as another woman explained: “All the supplies, a little bed for attending births, everything they need. They would have the roots, their plants, their garden with all their plants—nacedera, carpintero, all those things—with the roots planted there” (OG22). Her words convey a request not only for a space, but also for recognition of the symbolic and material environment that sustains midwifery care.

Figure 3 shows the entrance to a midwife’s home surrounded by medicinal plants that embody the connection between knowledge and the land. The image represents the ideal of a holistic home: a place for gathering, exchanging knowledge, and carrying out their work as guardians of life. However, this aspiration remains unrecognized by many health care institutions, which continue to exclude midwives from the system.

**Figure 3:**
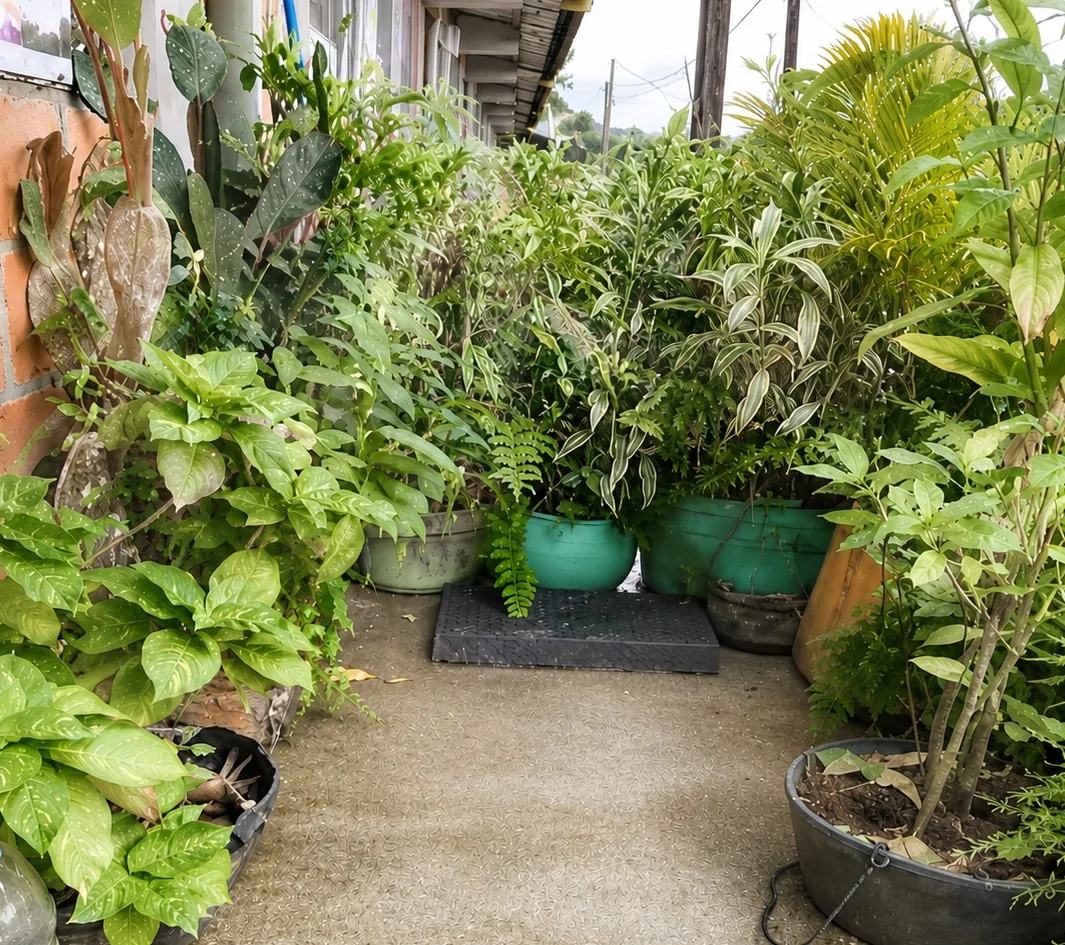
Front walkway of a midwife’s home in Nuquí

*Note: Photograph taken in 2023. It shows the front walkway—the outdoor area near the entrance—of a midwife’s home. As she leaves to carry out her work, she has medicinal plants readily available to treat the evil eye, including santa maría de anís (Piper auritum), yerba santa (Absinthium L.), doradilla (Selaginella lepidophylla), and desbaratadora (Justicia spicigera)*

Official recognition of midwives is also an act of social justice. Legitimizing their role within the health system can empower them, give them a voice and greater visibility in collective health decision-making, and strengthen the social fabric of their communities. This process should be accompanied by policies that support their practices and educational programs that recognize midwifery as a safe, accessible, and culturally appropriate option.

To advance this recognition, studies documenting the impact of midwifery on maternal and child health are essential. Indicators such as reductions in mortality rates and childbirth-related complications, as well as maternal satisfaction, can demonstrate the effectiveness of midwifery care. Qualitative studies that capture the voices of the women who received this care can further highlight the humane, emotional, and spiritual quality of the support provided. Such data are essential for raising awareness among health authorities and advancing the formal inclusion of midwifery.

However, this process of legitimation faces major obstacles. In many territories, the biomedical system continues to dismiss midwives’ knowledge, rendering it invisible within institutions and denying it legitimacy. In communities where access to health care services is limited, midwives serve as frontline care providers during pregnancy and childbirth.

Despite their experience and ability to recognize warning signs, midwives’ knowledge is often disregarded by health care personnel. “Look, this happened to me at the hospital itself, right there in Nuquí... I told the doctor, ‘The baby girl is already coming.’ But he did not listen to me. By the time he came, it was too late—the baby girl had died” (AM7). This account reveals the painful consequences of a lack of institutional recognition, which puts lives at risk and undermines collective work.

Institutional abandonment not only excludes midwives but also seeks to control the knowledge they have preserved for generations. This devaluation of their role also constitutes a way of imposing a single model of care that has historically marginalized women’s and community-based knowledge. “We midwives do not have supplies, and we are not recognized, but we are still here because we often save lives” (IV5), one woman stated, underscoring that their commitment to life persists despite the barriers imposed by the system.

Despite these adversities, midwives continue to dream of having a space of their own where they can practice their knowledge without depending on institutions that have marginalized them. “I dream of having a place of our own—a house where we could receive mothers and have everything organized. That would make things easier” (PM5). This aspiration, shared by many, reflects their desire for dignity and autonomy, as well as the need to continue caring for women in a setting that respects their roots.

Figure 4 shows a home that is more than a place of shelter; it is a symbol of resistance, knowledge, and care. It embodies the hope for a future in which midwives are recognized not as occasional assistants, but as central figures in the care and preservation of life.

**Figure 4:**
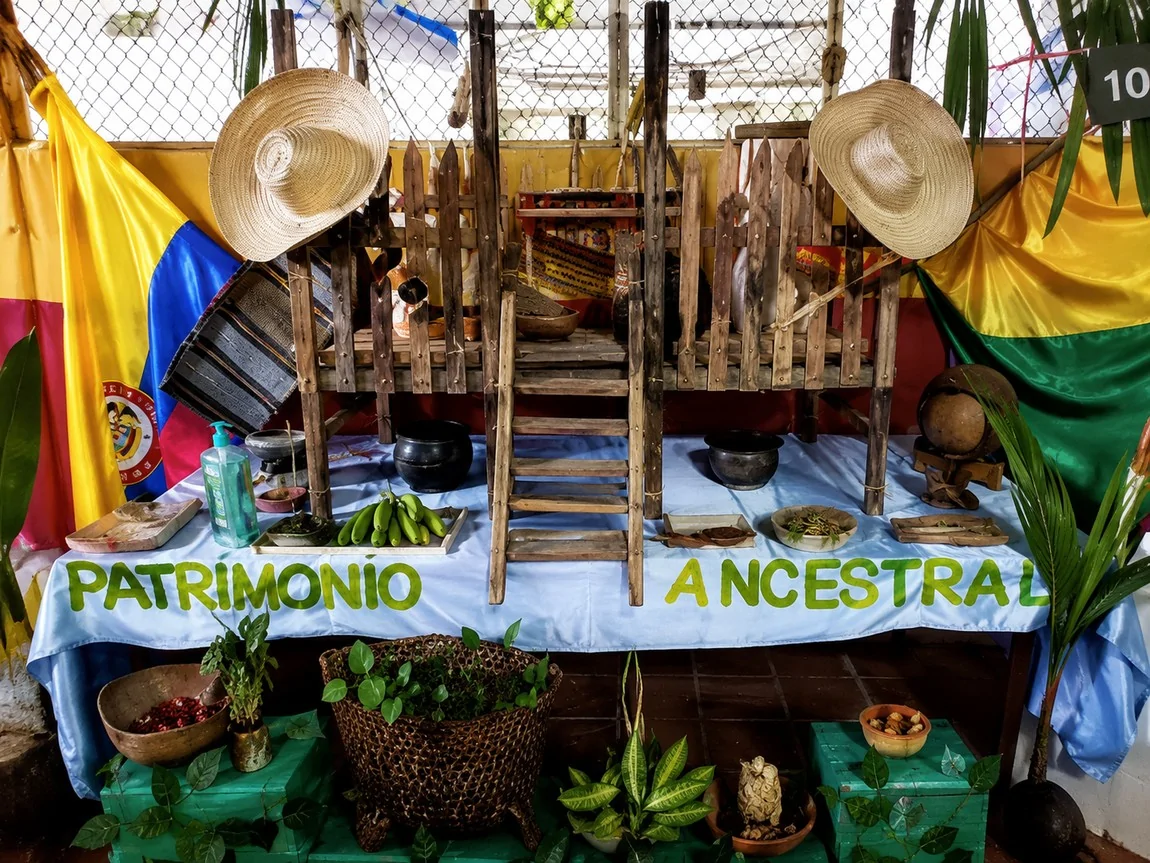
Home of an Asoparupa Midwife, 2021

*Note: Photograph provided by Asoparupa in 2021. It represents a dedicated space and the midwives’ dream of having a home equipped with the supplies needed to attend births. It also symbolizes ancestral heritage and territory*

In conclusion, legitimizing midwives’ knowledge is an urgent task that seeks not only to integrate different forms of knowledge but also to address historical patterns of exclusion. In the face of institutional abandonment and the control of knowledge, midwives continue to defend their practices as legitimate, effective, and profoundly humane ways of supporting childbirth. They are women who resist, care, and teach from the land, while claiming a dignified place within health systems and in the collective memory of their communities.

Taken together, the categories “practice and situated knowledge,” “free bodies and knowledge of the land,” and “legitimation of knowledge and grassroots power” provide an understanding of Afro-descendant midwifery as a comprehensive system of care deeply rooted in territory, memory, and women’s collective experience. Far from being a marginal practice, midwifery emerges as a form of cultural and political resistance that protects life, affirms the dignity of ancestral knowledge, and challenges the hegemonic logics of the biomedical system. These categories demonstrate how midwifery knowledge is not only transmitted and transformed in everyday and community settings but also asserted as a legitimate voice in the face of historical inequalities, reaffirming autonomy, grassroots power, and a vital connection to the land.

## Discussion

Afro-descendant and Indigenous communities have historically faced processes of dispossession rooted in colonial, patriarchal, and capitalist structures. However, they have not been passive victims; rather, they have acted as agents of resistance through their ancestral knowledge, spiritual practices, and connections to their territories. Within this context, women play leading roles as community leaders, caregivers, and guardians of collective memory, advancing transformative cultural, political, and epistemic initiatives.

From the category of “practice and situated knowledge,” women can be seen reinterpreting and reaffirming their knowledge through lived experience and dialogue with their surroundings. The Special Safeguarding Plan (PES)^23^ recognizes that midwives in Colombia’s Pacific region serve not only a caregiving role but also act as guardians of traditional knowledge, bringing together medicinal plants, spirituality, and community care in defense of their territory. This living body of knowledge, deeply connected to spirituality, becomes a central form of resistance to structural violence.

Several qualitative and ethnographic studies have shown that midwifery knowledge is acquired and applied through everyday practice, observation, and intergenerational transmission in domestic and community settings, shaping an embodied and context-specific body of knowledge that integrates the bodily, relational, and territorial dimensions of care^24^-^28^.

According to Lozano^29^, this spirituality brings social struggles together and strengthens leadership, while Lozano and Viveros^30^ highlight the symbiotic relationship among body, land, and spirituality as the foundation of a situated epistemology.

The category “free bodies and knowledge of the land” highlights the body as a political territory. Grueso^31^ and Gelacio et al.^32^ argue that Afro-descendant women reclaim and reinterpret their bodies as sites of memory and dignity, challenging extractivist, racist, and patriarchal narratives. This bodily reclamation is shaped through everyday life and in connection with nature. Along these same lines, movements advocating respectful maternity care and sexual and reproductive health demonstrate how the pregnant body becomes a site of agency in the face of practices of control, discipline, and obstetric violence, affirming the right to make decisions about one’s own body and the process of giving birth^33^.

Montañez Pico^34^ underscores the role of spirituality in fostering social cohesion amid crises generated by racial capitalism and cultural homogenization. Within midwifery practices, this spirituality and bodily autonomy are expressed through approaches to care that integrate the use of heat, medicinal plants, and relationships with the surrounding environment, giving rise to forms of knowledge that connect body, nature, and power through nonbiomedical logics^35^,^36^.

Ethnobotanical studies have shown that knowledge of medicinal plants is a central component of land-based knowledge, transmitted across generations and closely linked to cultural identity, language, and the defense of territory^37^,^38^. According to Vergara-Figueroa et al.^39^, women’s networks are essential for the intergenerational transmission of knowledge and the coordination of collective struggles.

From the perspective of “legitimation of knowledge and grassroots power,” the findings reveal the processes through which these women position their knowledge within institutional settings while contesting hegemonic frameworks. In maternal health, this contestation is reflected in the tension between global discourses that promote biomedical standards of “safe childbirth” and place-based midwifery practices, thereby producing epistemic hierarchies that subordinate community-based knowledge. Within this context, legitimation becomes a contested arena in which decisions are made about which forms of knowledge are recognized, who is authorized to practice them, and under what conditions^40^,^41^.

Crenshaw’s intersectional framework^42^ provides insight into how these women confront multiple, simultaneous forms of oppression while developing complex and creative responses that give rise to new ways of engaging in politics, without idealizing the past or abandoning their ethical and community-based principles.

Lugones^43^ argues that cultural differences can function as political tools. In this sense, Afro-descendant women advance transformative proposals from the local to the global level, engaging with decolonial feminisms and epistemologies of the South. Authors such as Collins et al.^11^ and Lugones^44^ emphasize the need to recognize the situated epistemologies of racialized women and advocate for a radical multiculturalism capable of transforming structures of exclusion and acknowledging their epistemic capacity.

With regard to traditional midwifery, this contestation is further complicated by ambivalent legal and policy frameworks that, while invoking cultural “dignification,” may also lead to mechanisms of control, the weakening of midwives’ role, and the risk of losing this cultural heritage. Legitimation, therefore, is not a neutral form of recognition but a process shaped by regulation, surveillance, and disputes over authority in care^45^. Likewise, recent experiences incorporating traditional midwives into institutional settings show that integration can create opportunities for recognition while also generating tensions arising from imposed protocols and power asymmetries. Even so, midwives employ strategies of negotiation and intercultural mediation that strengthen cultural safety and community care “from below,” thereby reconfiguring legitimacy through everyday practice^46^,^47^.

In urban settings, as Viveros Vigoya^48^ shows, Black middle-class communities reinterpret their ancestral knowledge in order to preserve cultural identity amid pressures of assimilation. This dialogue between ancestral and modern ways of knowing gives rise to evolving forms of resistance. Finally, according to Lozano and Grueso^49^, collective memory becomes a political resource that strengthens identity, supports claims for rights, and helps envision a more just future.

In sum, the three analytical categories provide insight into how Afro-descendant and Indigenous women reconfigure knowledge, bodies, and territories from a transformative perspective. Their leading role in defending ancestral knowledge, reclaiming and reinterpreting the body, and building grassroots power networks constitutes a political commitment to social and epistemic justice, reaffirming them as historical agents who forge pathways toward dignity, memory, and freedom.

## Conclusions

Traditional Afro-descendant midwifery constitutes a comprehensive system of care that brings together the body, territory, and collective memory. Grounded in ancestral knowledge and a profound relationship with nature, this practice challenges institutional neglect and biomedical hegemony while asserting autonomous and culturally situated ways of supporting life. Medicinal plants, community ties, and the intergenerational transmission of knowledge are fundamental pillars of this practice.

Within this framework, future research is needed to evaluate the impact of midwife-provided care on maternal and neonatal health and to further examine the role of health professionals, particularly nurses, as intercultural mediators capable of reducing tensions between the hospital-based biomedical system and traditional practices. Likewise, advancing certification and training, and integrating midwives into national health systems through intercultural and noncoercive approaches, is essential to strengthening their recognition.

Recognizing midwives entails not only including them in health systems but also committing to epistemic justice and to communities’ sovereignty over their ways of giving birth and providing care. Far from being a practice of the past, midwifery embodies a living form of resistance to medicalization and a political vision of dignity, equity, and grassroots power emerging from the margins.
